# Familial CJD Associated PrP Mutants within Transmembrane Region Induced Ctm-PrP Retention in ER and Triggered Apoptosis by ER Stress in SH-SY5Y Cells

**DOI:** 10.1371/journal.pone.0014602

**Published:** 2011-01-27

**Authors:** Xin Wang, Qi Shi, Kun Xu, Chen Gao, Cao Chen, Xiao-Li Li, Gui-Rong Wang, Chan Tian, Jun Han, Xiao-Ping Dong

**Affiliations:** 1 State Key Laboratory for Infectious Disease Prevention and Control, National Institute for Viral Disease Control and Prevention, Chinese Center for Disease Control and Prevention, Beijing, People's Republic of China; 2 School of Medicine, Xi'an Jiao-Tong University, Xi'an, People's Republic of China; University of North Dakota, United States of America

## Abstract

**Background:**

Genetic prion diseases are linked to point and inserted mutations in the prion protein (PrP) gene that are presumed to favor conversion of the cellular isoform of PrP (PrP^C^) to the pathogenic one (PrP^Sc^). The pathogenic mechanisms and the subcellular sites of the conversion are not completely understood. Here we introduce several *PRNP* gene mutations (such as, PrP-KDEL, PrP-3AV, PrP-A117V, PrP-G114V, PrP-P102L and PrP-E200K) into the cultured cells in order to explore the pathogenic mechanism of familial prion disease.

**Methodology/Principal Findings:**

To address the roles of aberrant retention of PrP in endoplasmic reticulum (ER), the recombinant plasmids expressing full-length human PrP tailed with an ER signal peptide at the COOH-terminal (PrP-KDEL) and PrP with three amino acids exchange in transmembrane region (PrP-3AV) were constructed. In the preparations of transient transfections, 18-kD COOH-terminal proteolytic resistant fragments (Ctm-PrP) were detected in the cells expressing PrP-KDEL and PrP-3AV. Analyses of the cell viabilities in the presences of tunicamycin and brefeldin A revealed that expressions of PrP-KDEL and PrP-3AV sensitized the transfected cells to ER stress stimuli. Western blots and RT-PCR identified the clear alternations of ER stress associated events in the cells expressing PrP-KDEL and PrP-3AV that induced ER mediated apoptosis by CHOP and capase-12 apoptosis pathway. Moreover, several familial CJD related PrP mutants were transiently introduced into the cultured cells. Only the mutants within the transmembrane region (G114V and A117V) induced the formation of Ctm-PrP and caused the ER stress, while the mutants outside the transmembrane region (P102L and E200K) failed.

**Conclusions/Significance:**

The data indicate that the retention of PrP in ER through formation of Ctm-PrP results in ER stress and cell apoptosis. The cytopathic activities caused by different familial CJD associated PrP mutants may vary, among them the mutants within the transmembrane region undergo an ER-stress mediated cell apoptosis.

## Introduction

Prion diseases, or transmissible spongiform encephalopathies (TSE), are a group of transmissible neurodegenerative disorders that afflict both humans and animals, including Creutzfeldt-Jakob disease (CJD) in human, scrapie in sheep and goat, bovine spongiform encephalopathy (BSE) in cattle[1]. The underlying cause of prion disease is the conversion of a host-derived cellular prion protein (PrP^C^) to the infectious scrapie prion protein (PrP^Sc^), a misfolded and proteinase K (PK)-resistant isoform, which is the main component of infectious prion[2], [3]. Propagation and accumulation of infectious PrP have always been thought to be tightly linked to the pathogenesis of prion disease. However, transgenic mouse models have revealed that misfolded or mistargeted PrP^C^ can also induce neuronal cell death in the absence of infectious prion[4]. The observation of substantial neurodegeneration in the absence of PrP^Sc^ accumulation in some cases of natural and experimental prion disease argues against its accumulation as the sole cause of pathology[5], [6], highlighting that other aspects, e.g. PrP expressing, folding, locating and trafficking, may feature in the pathophysiological mechanisms that ultimately cause disease.

Studies of PrP translocation at the ER membrane have revealed unusual features in its biogenesis. Cell-free translation systems have figured out more than one topologic form[7], [8], one of which appears to be fully translocated into the ER lumen, termed the secretory form (Sec-PrP). This topology is much more similar to the PrP^C^, which is on the cell surface, adhered to the membrane by a glycolipid anchor. Two other distinct topologic forms of PrP have been found in ER: one is fully translocating that has the COOH-terminus in the ER lumen with the NH_2_-terminus accessible to proteases in the cytosol, termed cytosol transmembrane form (Ctm-PrP)[7], [8]; the other form, termed N-terminal transmembrane (Ntm-PrP), has the NH_2_-terminus in the ER lumen with the COOH-terminus accessible to proteases in the cytosol, which has been confirmed no cytotoxicity affection to the cells[9].

It has been described that the specific Ctm-PrP confers severe neurodegeneration in the experimental mice with features of typical prion disease[9]. In some cellular models of inherited prion disorders, misfolded mutant prion proteins accumulate in the ER and activate the ER stress cytotoxic response, resulting in neuronal cell death[10]. Retrotransportion of PrP^C^ from trans-Golgi compartment toward the ER increases the production of PrP^Sc^ in persistently scrapie-infected neuroblastoma N2a cells[11].

Ctm-PrP appears to span the membrane at the same hydrophobic stretch in PrP (roughly residues 112 to 135, refers to TM1). Human PrP comprise 253 aa including N-terminal signal sequence (from aa 1 to 22), C-terminal GPI sequence (from aa 231 to 253) and the hydrophobic transmembrane region. Some specific mutations in this region have been reported to be related with human genetic or familial CJD (fCJD). In 1995, an A117V mutation [alanine (A) to valine (V) substitution at position 117] was described to be associated with fCJD in two kindreds[12]. Another fCJD-related mutant, G114V, [glycine (G) to valine (V) substitution at position 114] was reported in a Uranian family[12] and lately in a Chinese family[13]. Whether these fCJD-associated PrP point mutations lead to formation Ctm-PrP in ER remain unknown.

Although previous data provide the evidences that PrP retained in ER induced cell death and prion disease in animals, the detailed processes still need to be elucidated. In this study, a four amino acids tag, which was previously confirmed to be a signal peptide for ER location, was added to the C-terminus of PrP (deleted the GPI sequence, aa 231–253, PrP-KDEL). Transient expression of this PrP resulted in the formation of Ctm-PrP in ER, and triggered ER stress and apoptosis, which was same as PrP-3AV[9]. Moreover, expressions of PrP mutants within transmembrane region, i.e. PrP-G114V and PrP-A117V, induced the similar effectiveness on the cultured cells as PrP-KDEL, while the PrP mutants outside the transmembrane region, i.e. PrP-P102L and PrP-E200K did not cause ER stress, in spite of apoptosis.

## Materials and Methods

### Plasmid construction

Human PrP gene encoding the amino acid (aa) 1–230 with a four amino acids (Lys-Asp-Glu-Leu) at its C-terminus (deleted aa231-253, PrP-KDEL) was amplified by PCR technique, using recombinant plasmid pcDNA3.1-huPrP1-253[14] as the template, with the forward primer (5′-ggatccatggcgaaccttggctgctg-3′, with BamH I site underlined) and reverse primer (5′-gcggccgcctagagctcatccttc tccgacttgtacagctcgctcat-3′, with Not I site underlined). The PCR product was cloned into vector pcDNA3.1(zeo+), generating plasmid pcDNA3.1-huPrP-KDEL. The mammalian expression plasmid pcDNA-huPrP-3AV, in which the alanine (Ala) at position 113, 115 and 118 were replaced by valine (Val), was generated by PCR technique using pcDNA3.1-PrP1-253 as the template. The mutated PrP gene was amplified by PCR with the forward upstream primer PrP-3AV-F (5′-ggatccatggcgaac cttggctgctggatg-3′, with a BamH Ι site underlined), and downstream primer PrP-3AV-R (5′-gcgaattctcatccca ctatcaggaagatga-3′, with a EcoR Ι site underlined), as well as internal primer PrP-3AV-1 (5′-tgccacagccactgcagccacagccat-3′) and PrP-3AV-2 (5′-atggctgtggctgcagtggctgtggca-3′), under the following conditions, 94°C for 30 s, 63°C for 30 s and 72°C for 30 s, totally 30 cycles. PCR products were inserted into vector pcDNA3.1(zeo+), yielding recombinant plasmid pcDNA3.1-huPrP-3AV.

**Figure 1:**
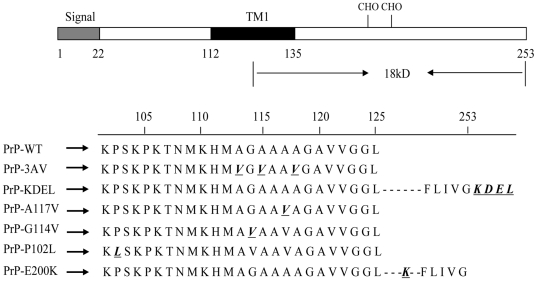
Scheme of the topogenic domains in PrP. Topogenic sequences shown are NH_2_-terminal signal sequence (signal), the potential membrane-spanning domain (TM1) and glycosylation site (CHO). Amino acid positions are shown below the diagram. The amino acid sequences of wild type and the mutated PrPs used in this study are illustrated below. The amino acid substitutions in PrP mutants are indicated by black, italic and underlined letter.

Mammalian expression plasmids containing the full-length human PrP genes (aa 1–253) of familial CJD (fCJD)-related PrP mutants, including P102L, G114V and E200K, were generated by PCR protocol using the extracted DNAs from individual Chinese patient's peripheral blood[13], [15], and subcloned into vector pcDNA3.1(zeo+), yielding recombinant plasmids pcDNA3.1-huPrP-P102L, pcDNA3.1-huPrP-G114V, and pcDNA3.1-huPrP-E200K. Plasmid pcDNA3.1-huPrP-A117V containing mutated PrP-A117V was constructed by a site-direction mutation PCR protocol, using pcDNA3.1-PrP1-253 as the template. Various recombinant human PrP expressing plasmids were illustrated in Fig. 1.

To construct mammalian expressing plasmid encoding the full-length presenilin 1 (PS1) with an extra amino acid KDEL at the C-terminus, the sequence of human PS1 gene was amplified from cDNA transcribed from human total RNA by a reverse transcriptional PCR (RT-PCR), using forward primer 5′-ggatccatgacaga gttacctgcaccgttg-3′ (with BamH I site underlined) and reverse primer 5′-gcggccgcctagagctcatccttctccgagatataaaattgatggaatgc-3′ (with Not I site underlined). The PCR product was cloned into vector pcDNA3.1(zeo+), generating recombinant plasmid pcDNA3.1-PS1-KDEL.

### Cell culture, transfection and proteinase K (PK) digestion

Human neuroblastoma cell line SH-SY5Y was maintained in DMEM (Gibco BRL, USA). Cells were plated into 6-well or 96-well plates (Falcon, Japan) 24 hr before transfection. Different amounts of plasmids (2 µg DNA per well in a 6-well plate and 0.2 µg DNA per well in a 96-well plate) were transfected into the monolayer cells with Lipofectamine™ 2000 transfection reagent (Invitrogen, USA). The transfection efficacy of each preparation was evaluated by counting the ratio of PrP positive stained cells. Cells were harvested by trypsin/EDTA in PBS 12 or 20 hr after transfection, pelleted by short centrifugation and suspended in the lysis buffer (10 mM Tris-HCl, pH 7.8, 0.5% sodiumdeodycholate, 0.5% Nonidet P-40, 100 mM NaCl, 10 mM EDTA), supplemented with complete proteasomal inhibitor mixture. For proteolysis experiments, 100 µg of cell lysates were incubated with proteinase K (PK) at various concentrations at 37°C for 30 min. The reaction was terminated by addition of Pefabloc SC (Roche), boiling in Laemmli sample buffer. Residual PrP signal was detected by PrP-specific Western blot.

### Western blot

The cellular lysates were separated by 15% SDS-PAGE and electro-transferred onto nitrocellulose membranes. After blocking with 5% nonfat-dried milk in PBST (phosphate buffered saline, pH 7.6, containing 0.05% Tween-20) overnight at 4°C, the membranes were incubated with 1∶4000 PrP specific monoclonal antibody (mAb) 3F4 (Dako, UK), 1∶1000 rabbit polyclonal antibody anti-human GRP78 (Santa Cruz, USA), 1∶1000 goat mAb anti-human CHOP and 1∶500 goat polyclonal antibody anti-human caspase-12 (Stressgen, USA), 1∶600 mAb anti-human β-actin (Santa Cruz, USA) and 1∶1000 polyclonal antibody pro-anti-human caspase-3 (Santa Cruz, USA) for 2 hr at room temperature, and then incubated with 1∶4000 horseradish peroxidase (HRP)-conjugated anti-mouse, anti-rabbit or anti-goat IgG (Santa Cruz, USA). The reactive signals were visualized by ECL kit (PE Applied Biosystems, Foster City, USA).

### PNGase F and Endoglycosidase (Endo H) treatment

Cells lysates were resuspended in denaturing buffer (0.5% SDS, 1% β-mercaptoethanol) and boiled for 10 min. Samples were deglycosylated with PNGase F (1000 units, New England Biolabs, in 1% Nonidet P-40, 50 µM sodium citrate, pH 7.5) or endoglycosidase H (1000 units, New England Biolabs, in 50 µM sodium citrate, pH 5.5) for 60 min at 37°C. Proteins were separated on 15% SDS acrylamide gels.

### Phosphatidylinositol-specific phospholipase C (PI-PLC) GPI cleavage assay

The confluent cultures of cells were washed with ice-cold PBS and incubated with PI-PLC (Sigma, USA) at a concentration of 60 ng/ml in DMEM (Gibco BRL, USA) for 1.5 hr at 37°C by shaking at 100 rpm. The medium was collected and centrifuged at 16,000×g for 5 min to remove loose cells, and proteins in the supernatant were precipitation with 4 volumes of methanol at −20°C for 4 hr. Precipitation proteins were collected by centrifugation at 16,000×g for 30 min at 4°C and resuspended in SDS-PAGE sample buffer. Cells pellets were lysed directly in ice-cold lysis buffer. PrP was visualized by SDS-PAGE and Western blotting with mAb 3F4.

### MTT analysis

Cells were cultured in 96-well tissue culture plates and transiently transfected with individual PrP expressing plasmids. Cell viability was assessed by the conversion of 3-(4, 5-Dimethylthiazol-2-yl)-2, 5-diphenyl-2H-tetrazolium bromide (MTT, Sigma, USA) to a formazan product. 12 to 24 hr after transfection, MTT was added to each well to a final concentration of 5 mg/ml in culture medium and incubated at 37°C for 4 hr. The reaction was terminated by removal of the supernatant and addition of 200 µl DMSO per well to dissolve the formazan product. The plates were read at 540 nm on a micro-ELISA plate reader (Thermo MK3, USA). Each assay was performed in duplication of at least four wells.

### Tunicamycin and brefeldin A resistant characteristics assay

2×10^4^ SH-SY5Y cells were grown in 96-well plates and treated with tunicamycin and brefeldin A for 12 and 20 hr at the final concentration of 10 µg/ml and 20 µg/ml, respectively. Cell viability was quantified using MTT analyses as above. The data collected from two independent experiments in triplicate. The resistant capabilities, nominated as data cell viability in MTT assays, of each preparation to tunicamycin or brefeldin A was the difference between the average value of the test with and without tunicamycin or brefeldin A.

### Ctm-PrP detection

For Ctm-PrP detection, a mild proteolysis conditions were conducted, which has been proved that PK could only access to the outside of the ER vesicles[9]. Cells were released from dishes by EDTA solution(0.02%) at 37°C in PBS and exposed to 0.25 mg/ml PK and 1% NP-40 for 60 min on ice. The proteolysis reactions were terminated by adding PMSF to 5 mM and incubated for an additional 5 min. The preparations were further mixed with 5 volumes of boiling buffer (1% SDS and 0.1 M Tris, pH 8.9), digested with PNGase F and transferred onto nitrocellulose and probed with mAb 3F4.

### RNA extraction and semi-quantitative RT-PCR

To measure transcriptional situations of the ER stress-associated agents in the tested SH-SY5Y cells, a set of semi-quantitative RT-PCR assays was designed, including Grp58, PERK, Bip, CHOP and calreticulum(table 1). The specific primers for Grp58, Perk, Bip, CHOP and calreticulum were synthesized according to the previous reports [16]. In parallel, the transcriptional situation of individual β-actin was measured as the internal control. With an RNAsimple Total RNA Kit (TIANGEN, China), total cellular RNA were prepared. Reverse transcription was performed using SuperScript™ III First-Strand Synthesis System (Invitrogen, USA). Briefly, 2 µg of total RNA was mixed with 200 U of MMLV reverse transcriptase and 50 pM oligo (dT_20_) in a volume of 20 µl. The mixtures were maintained at 50°C for 50 min and inactivated by heating at 85°C for 5 min. To remove the RNA from the cDNA, 1 µl RNase H was added to the mixture and incubated at 37°C for 20 min. Aliquots (2 µl) of RT reaction products were amplified by PCR(the Taq used with proofreading activity) in a volume of 50 µl under the following conditions: 94°C for 40 s, 60°C for 30 s and 72°C for 30 s, totally 30 cycles. After electrophoresed on 1.4% agarose gels, the gel images of each PCR product were digitally captured with a CCD camera and analyzed with the NIH Imager beta version 2. Relative transcriptional values of each factor in hemi-quantitative RT-PCR are presented as a ratio of the signal value of the specific PCR product and that of the individual β-actin.

**Table 1:** The sequences of the primers used in the semi-quantitative RT-PCR analysis for mRNA levels of the ER-stress related genes.

Gene	Primer Sequences	
β-actin	Forward	GGACTTCGAGCAGGAGATGG
	Reverse	GCACCGTGTTGGCGTAGAGG
Bip	Forward	TCATCGGACGCACTTGGAA
	Reverse	CAACCACCTTGAATGGCAAGA
Calreticulin	Forward	TTACGCACTGTCCGCCAAA
	Reverse	GCTCATGCTTCACCGTGAACT
Grp58	Forward	TCAAGGGTTTTCCTACCATCTACTTC
	Reverse	TTAATTCACGGCCACCTTCAT
PERK	Forward	AAGTAGATGACTGCAATTACGCTATCAA
	Reverse	TTTAACTTCCCGCATTACCTTCTC
CHOP	Forward	GTCCCTAGCTTGGCTGACAGA
	Reverse	TGGAGAGCGAGGGCTTTG

### Caspase-3 activity

Caspase-3 activity was measured according to a protocol described as manufacturer (Sigma, A2559, USA). Briefly, Ac-DEVE-pNA was used as the substrate for activated caspase-3, and typically 1-2×10^6^ cells were used. Reactions were conducted in 96 well plates at 37°C for 2 hr in the dark. Absorbance was determined with a microELISA plate reader (Thermo MK3, USA) at 405 nm.

### Annexin V-FITC binding

Cell viability was assessed by flow cytometry that monitored annexin V-FITC binding and propidium iodide (PI) (Trevigen, USA) uptake simultaneously. Briefly, 12 or 20 hr after transfection, cells were harvested and resuspended in annexin V-FITC binding buffer and incubated with annexinV-FITC (1×) and PI (5 µg/ml) in the dark at room temperature for 15 min. Samples were analyzed on a FACScan flow cytometry (Becton Dickinson, Oxford, UK). Fluorescence was measured through a 530/30 band filter (FL-1) to monitor annexin V-FITC binding and through a 585/42 band filter (FL-2) to monitor PI uptake.

### Statistic analysis

Quantitative analysis of immunoblot images was carried out using computer-assisted software Image Total Tech (Pharmacia, USA). Briefly, the image of immunoblot was scanned with Typhoon (Pharmacia, USA) and digitalized, saved as TIF format. The values of each target blot were evaluated. All data are presented as the mean ± SD. Statistical analysis was performed using the *T* test. Probabilities of less than 0.05 were considered to be statistically significant.

## Results

### Transiently expressed PrP-KDEL and PrP-3AV partially retained in ER of the cells

**Figure 2:**
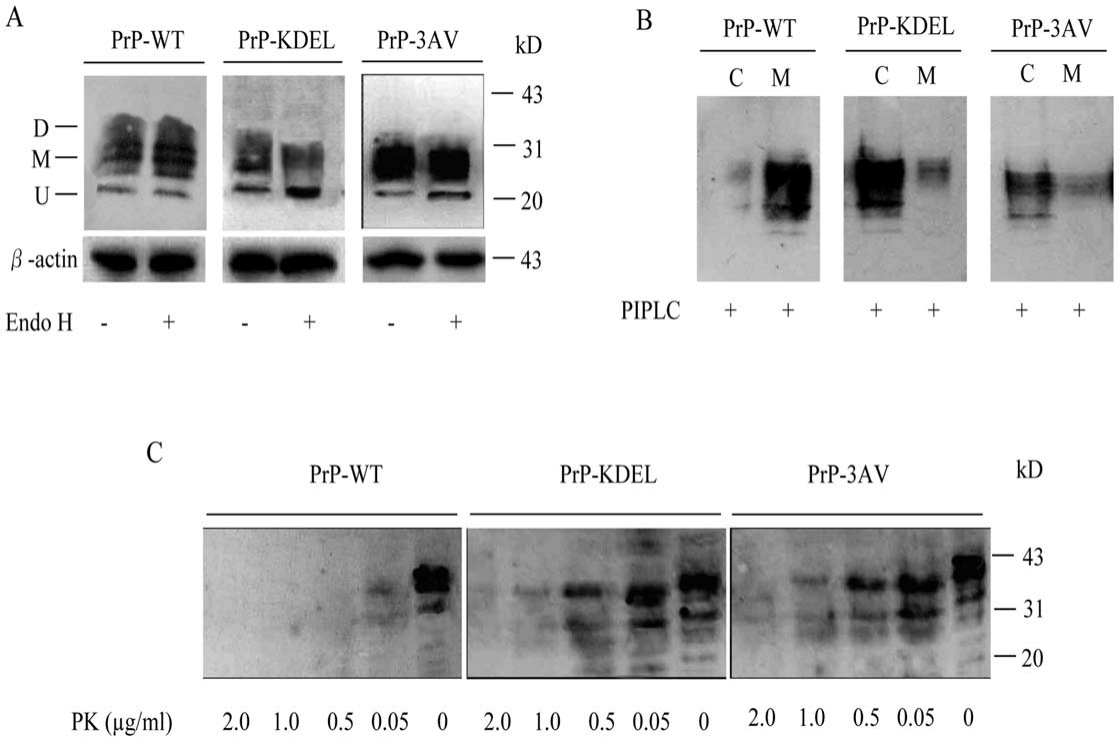
The expressive and biochemical profiles of WT- and mutated-PrPs expressed in SH-SY5Y cells. A. Endo digestion. After digested with Endo H (shown in below), the samples were analyzed by immunoblots with PrP specific mAb 3F4. “D” refers to diglycosylated, “M” refers to monoglycosylated and “U” refers to unglycosylated PrPs, respectively. The positions of D, M and U are indicated on left and molecular size markers are indicated on right. B. PI-PLC resistance. Cells expressing WT- and mutated-PrPs were incubated with PI-PLC for 1.5 hr at 37°C, and then PrP signals in the fractions of medium (lane M) and cellular lysates (lane C) were revealed by Western blots with mAb 3F4. C. PK-resistance. Lysates of cells expressing WT- and mutated-PrPs (100 µg of total protein) were treated with the different concentrations of PK (shown in below) at 37°C for 30 min, and PrP signals were detected by Western blots with mAb 3F4.

Enzyme endoglycosidase H (Endo H) has the ability to remove carbohydrates of the proteins retained in the ER after expression [17]. Three expressing plasmids, PrP-WT, PrP-KDEL and PrP-3VA, were transiently transfected into SHSY5Y cells. PrP-specific Western blots revealed three PrP specific signals from 25 to 35 kDa in the cells expressing PrP-WT, which represented di- mono- and un-glycosylated PrP. Similar pattern was detected in the cells expressing PrP-3AV, whereas predominant un-glycosylated PrP in the cells expressing PrP-KDEL (Fig. 2A). After digestion of Endo H, the PrP profiles of the cells expressing PrP-WT were almost unchanged, while that of the cells expressing PrP-3AV showed slightly but repeatedly identified increased un-glycosylated PrP (Fig. 2A). The PrP profile of the cells expressing PrP- KDEL were totally different, that the signals of di- and mono-glycosylated PrP decreased significantly, and that of un-glycosylated PrP increased (Fig. 2A).

To test the potential differences in the distributions of the expressed PrPs, the cells were employed to the digestion of the bacterial enzyme PI-PLC that cleaves the C-terminal GPI anchor 24 hr after transfection[18]. After centrifugation, the PrP amounts of the fractions of supernatant and pellet were separately evaluated by Western blots. It showed that most portion of PrP-WT distributed in the fraction of supernatant, contrarily, large portion of PrP-3AV and PrP-KEDL in the pellets (Fig. 2B). Interestingly, almost all mono- and un-glycosylated PrP in the cells expressing PrP-3AV and PrP-KDEL located in the fractions of pellets. These results highlight that under the experimental situation PrP-WT undergoes normal process posttranslationally and localizes mostly on the surface of the cells, while majorities of PrP-3AV and PrP-KDEL are in the intra-cytoplasm.

To evaluate the PK-resistances of the PrP mutants expressed in the cells, same amounts of the cell lysates were subjected to various concentrations of PK treatment. Clear PrP signals were still identified in the preparation of PrP-KDEL treated with 1.0 µg/ml PK and that of PrP-3AV treated with 0.5 µg/ml PK, while only observed in the preparation of PrP-WT treated with 0.05 µg/ml PK (Fig. 2C), indicating that the expressed PrP mutants possess stronger PK resistance.

**Figure 3:**
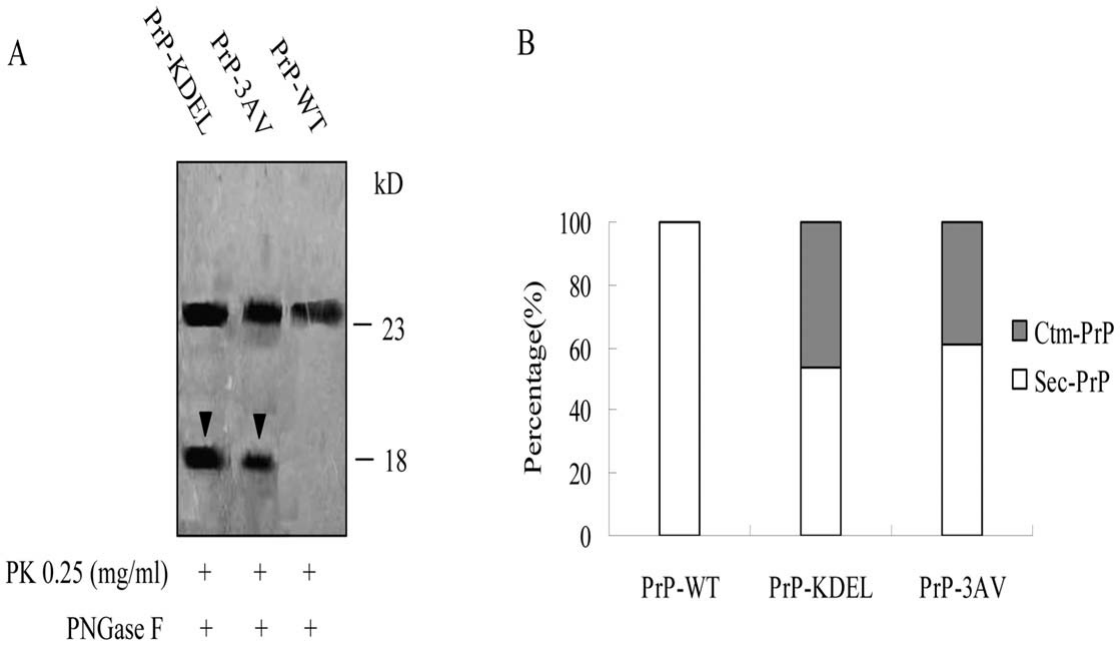
Formations of Ctm-PrP in the cells expressing WT- and mutated-PrP. A. The suspended cells were subjected to digestion with 0.25 mg/ml PK (mild PK digestion). After terminating the proteolysis reaction, samples were digested with PNGase F and analyzed by SDS-PAGE and immunoblots with mAb 3F4. The arrowheads in lanes point to the Ctm-PrP form. B. Quantitative representations of the relative amounts of Sec-PrP (mature PrP, white bars) and Ctm-PrP (black bars) for PrP-KDEL and PrP-3AV.

To address formation of Ctm-PrP in the transfected cells, a mild proteolysis process followed by PNGase F digestion was performed, which may allow producing an 18-kDa PrP C-terminal fragment (9). Prior to the mild proteolysis process, the cell lysates transiently expressing PrP-WT, PrP-KDEL and PrP3VA, as well as that treated with PNGase F, were confirmed to have similar reactive patterns in Western blots (Fig. S1). After treatments with mild proteolysis of PK and PNGase F, a roughly 25 kDa band was observed in the preparation of PrP-WT, which represented deglycosylated PrP molecule (Fig. 3A). In addition to the 25 kDa band, another 18 kDa PrP-specific band was detected in the preparations of PrP-KDEL and PrP-3AV (Fig. 3A), which occupied about 46% and 39% of total PrPs after digitalizing the signal intensities (Fig. 3B). Compared with that of PrP-3AV, expression of PrP-KDEL induced more formation of Ctm-PrP.

### Expressions of PrP-KDEL and PrP-3AV sensitized the transfected cells to ER stress stimuli

**Figure 4:**
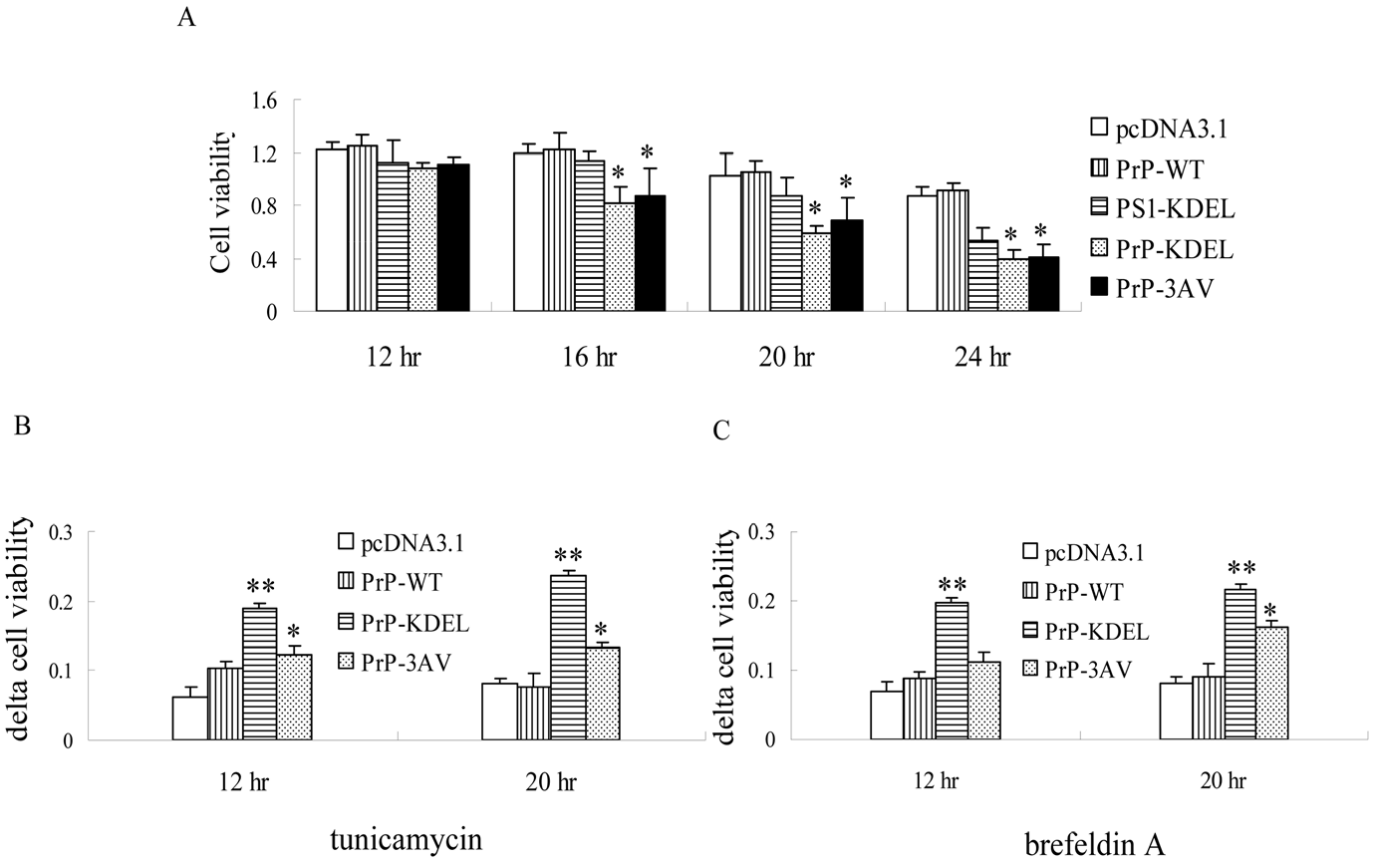
Influences of the ER stress stimuli on the cell viability measured by MTT assays. A. The cell viabilities of each preparation 12, 16, 20 and 24 hours post-transfection. B and C. The resistant capabilities (delta cell viability) of the cells expressing PrP-WT and PrP mutants treated with tunicamycin (B) and brefeldin A (C). Statistical differences of the data of each preparation compared with that of WT-PrP were illustrated as * *P<0.05* and ** *P<0.01*, respectively. The average data of each preparation was calculated based on three independent experiments and represented as mean ± SD.

To see the influence of the Ctm-PrP retained in ER on the growth abilities of the cultured cells, the cell viabilities were measured with MTT analyses 12, 16, 20 and 24 hr after transfection, respectively. In parallel, a recombinant plasmid pcNDA3.1-PS1-KDEL that expressed presenilin 1 protein in ER was used as control, in order to exclude the potential influence caused by the KDEL-tailed protein. MTT assays of the preparations of 12 hr after transfection revealed no difference in the cell viabilities among various groups. However, in the preparations of 16 hr after transfection, expressions of PrP-KDEL and PrP-3AV resulted in significantly lower cell viabilities compared with that of PrP-WT (*P*<0.05, Fig. 4A). In addition, expressions of PS1-KDEL did not decrease the cell viabilities in the reactions 12 and 16 hr after transfection, while showed lower MTT values in the preparations 20 and 24 hr but without significant difference compared with the controls (Fig. 4A).

The cells receiving the recombinant plasmids expressing PrP-KDEL, PrP-3AV and PrP-WT were exposed to different concentrations of brefeldin A and tunicamycin and their cell viabilities were evaluated by MTT assays 12 and 20 hr after transfection. As we expected, transfection of plasmid expressing PrP-WT resulted in similar delta cell viability as the mock (pcDNA3.1, Fig. 4B and 4C) after challenge. The delta cell viability of the cells expressing PrP-KDEL obviously increased in the presences of brefeldin A (4B) and tunicamycin (4C), showing significantly statistic difference compared with that of PrP-WT (*P<*0.01). Expression of PrP-3AV also induced higher delta cell viability in the presences of brefeldin A and tunicamycin, compared with that of PrP-WT (*P<*0.05, Fig. 4B and 4C). Both PrP-KDEL and PrP-3AV groups showed statistically lower cell viabilities at early stage (12 hr after transfection) after challenge, implying that impairing ER normal function by mislocation of Ctm-PrP in ER happens earlier than causing cell death (16 hr after transfection, Fig. 4A).

### Presence of Ctm-PrP in the cells triggered the ER stress

**Figure 5:**
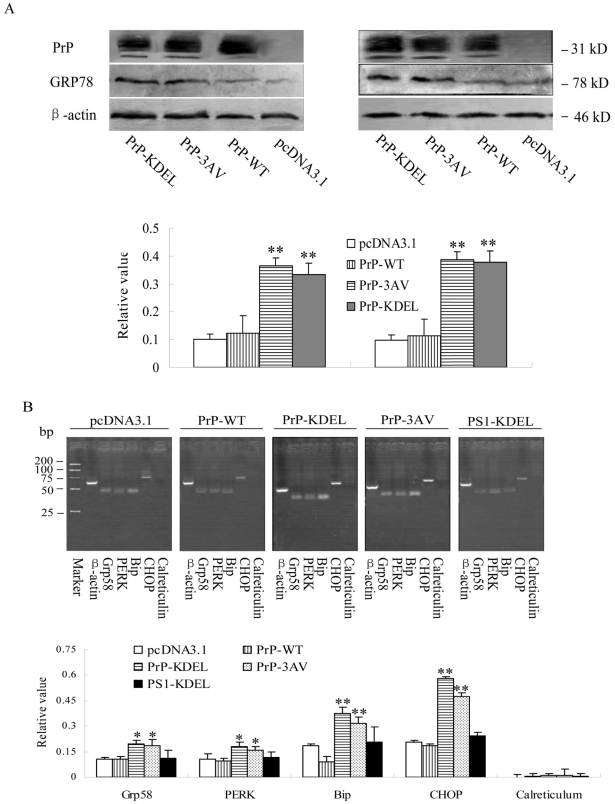
Alterations of ER stress related events in the cells expressing WT- and mutated PrPs. A. Analyses of the levels of ER stress associated protein Grp78 in SH-SY5Y cells 12 (left) and 20 hr (right) after transfection by Western blots. The average gray value of each preparation was calculated by the gray numerical value of Grp78 vs that of β-actin. The average data of each preparation was evaluated based on three independent blots and represented as mean ± SD. Statistical differences of the data of each preparation compared with that of WT-PrP were illustrated as * *P*<0.05 and ** *P*<0.01, respectively. B. Semi-quantification of mRNA levels of the ER stress associated genes in the cells 12 hr after transfection by RT-PCR PCR products of CHOP (55 bp), Bip (54 bp), Grp58/ERp57 (51 bp), PERK (72 bp) and calreticulin (54 bp) were shown in 1.5% agarose gels above. The relative value of each preparation was calculated by the gray numerical value of each specific product vs that of β-actin (61 bp). The average data of each preparation was evaluated based on three independent reactions and represented as mean ± SD. Statistical differences of the data of each preparation compared with that of PrP-WT were illustrated as * *P*<0.05 and ** P<0.01, respectively.

Grp78 has been recognized as an ER stress marker[19]. To see the possible change of Grp78 after expressions of Ctm-PrP, the cells expressing PrP-WT, PrP-KDEL and PrP-3AV were harvested 12 and 20 hr after transfection and the cellular Grp78 were determined by Western blots. Fig. 5A revealed that the levels of Grp78 in the preparations of PrP-KDEL and PrP-3AV were higher than that of PrP-WT and mock (pcDNA3.1) 12 hr after transfection, showing significant statistic difference (*P<*0.01) after digitalizing the individual signal intensities with the values of β-actin. 20 hr after transfection, the levels of Grp78 in all preparations take on the same tendency as 12 hr post-transfection (Fig. 5A). It suggests that the presence of Ctm-PrP triggers ER stress at early stage.

To get more data of ER stress, the transcriptional levels of several other agents related with ER stress, including CHOP, Bip, Grp58 and PERK and calreticulin were analyzed with semi-quantity RT-PCR 12 hr after transfection. The signal intensity of each PCR product was colleted by a computer-assistant scanner and the relative value was calculated by equilibrating the signal intensity of individual β-actin PCR product. As shown in Fig. 5B, the mRNA levels of CHOP, PERK, Bip, Grp58 were increased in the preparations of PrP-KDEL and PrP-3AV, revealing statistic difference compared with PrP-WT and mock (pcDNA3.1). Additionally, the levels of calreticulin remained comparable among the preparations (Fig. 5B). These data strongly indicate emergence of an ER stress after expression Ctm-PrP.

### Ctm-PrP induced ER stress further mediated the cell apoptosis

**Figure 6:**
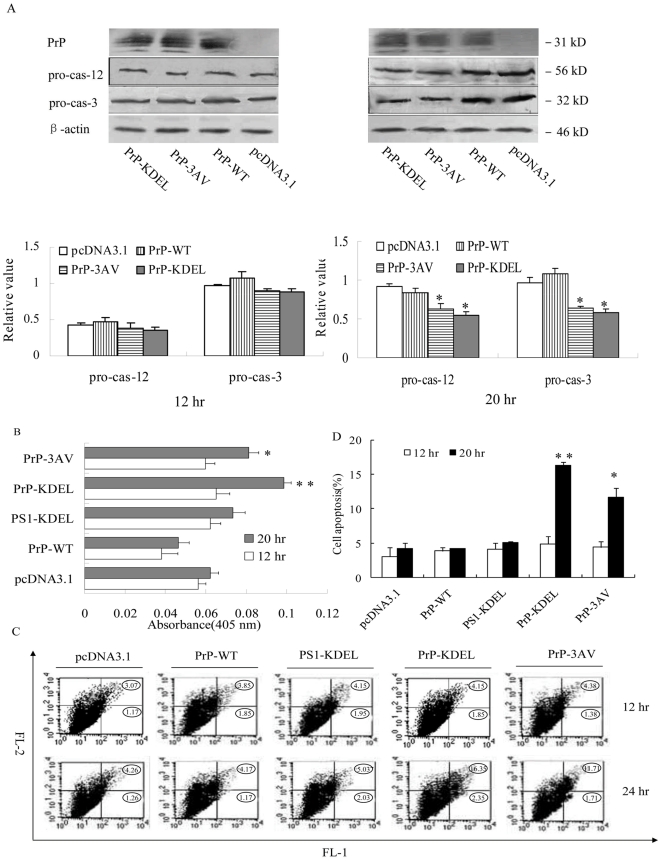
Alterations of apoptosis related agents in the cells expressing WT- and mutated-PrPs. A. Levels of pro-caspase 12 and pro-caspase 3 in the cells 12 (left) and 20 (right) hr post-transfection with Western blots. The average gray value of each preparation was calculated by the gray numerical value of pro-caspase-12 or pro-caspase-3 vs that of β-actin. The average data of each preparation was evaluated based on three independent blots and represented as mean ± SD. Statistical differences of the data of each preparation compared with that of WT-PrP were illustrated as * *P*<0.05 and ** *P*<0.01, respectively. B. Total cellular caspase activities of the cells 12 (white column) and 20 (grey column) hr post-transfection. Absorbance value (450 nm) of each reaction was measured by a microELISA plate reader. The average data of each preparation was calculated based on three independent experiments and represented as mean ± SD. * indicating *P*<0.05, ** indicating *P*<0.01. C. Annexin V-FITC/PI assays of the cells 12 and 20 hr post-transfection. Apoptosis was measured by annexin V-FITC binding via flow cytometry (represented by an increase in FL-1). Secondary necrosis is indicated by a subsequent increase in FL-2. The percentage of the overall population in each quadrant is given in the circles. D. Quantitative graphs of annexin V-FITC/PI staining. The average percentage of apoptosis cells were calculated from three independent assays and represented as mean ± SD. Statistical differences of the data of each preparation compared with that of PrP-WT were illustrated as * *P*<0.05.

The caspase levels and activities of the cells receiving the plasmids expressing PrP-KDEL, PrP-3AV, PrP-WT and PS1-KDEL were tested 12 and 20 hr after transfection. Western blots of pro-caspase-12 and pro-caspase-3 identified that in the preparations of 12 hr after transfection the levels of those two factors were comparable, although that of PrP-KDEL and PrP-3AV were slightly lower than that of PrP-WT and mock (pcDNA3.1) without statistic difference, whereas in the preparations of 20 hr after transfection the levels of both factors significantly reduced in the cells expressing PrP-KDEL and PrP-3AV (Fig. 6A). In line with these observations, in the preparations of 12 hr after transfection the total cellular caspase activities of the cells expressing PrP-KDEL and PrP-3AV were quite similar as that of the mock, even PrP-WT group showing lower activity than mock (Fig. 6B). 20 hr after transfection, the cells expressed PrP-KDEL and PrP-3AV showed significantly higher caspase activities (*P<*0.05 in PrP-3AV and P*<*0.01 in PrP-KDEL, Fig. 6B). Additionally, the caspase activities of the cells expressing PS1-KDEL did not revealed statistic difference compared with the mock (Fig. 6B). These results suggest that followed CHOP pathway, the caspase apoptosis pathway was activated after ER stress due to Ctm-PrP accumulation.

To figure out more apoptosis events, cells expressing various PrPs and PS1-KDEL were tested by flow cytometry with annexin V/PI staining. In the preparations 12 hr after transfection, the amounts of annexin V-FITC-positive stained cells represented the apoptotic population (low right quadrant) varied little among various preparations (Fig. 6C). In the preparations of 20 hr after transfection, the percentages of annexin V-FITC-positive stained cells were obviously increased in the groups of PrP-KDEL and PrP-3AV, while that in the group of PS1-KDEL (upper right quadrant) were almost same as that in the controls (Fig. 6C). Calculations of the average percentages of apoptostic cells positively stained with V-FITC in the cells expressing PrP-KDEL and PrP-3AV 20 hr after transfection showed statistical difference compared with that in groups of PrP-WT and mock (*P<*0.05, Fig. 6D). It implies that cell apoptosis was induced by the Ctm-PrP-associated ER stress.

### The natural familial CJD associated PrP mutants in transmembrane region formed the Ctm-PrP

**Figure 7:**
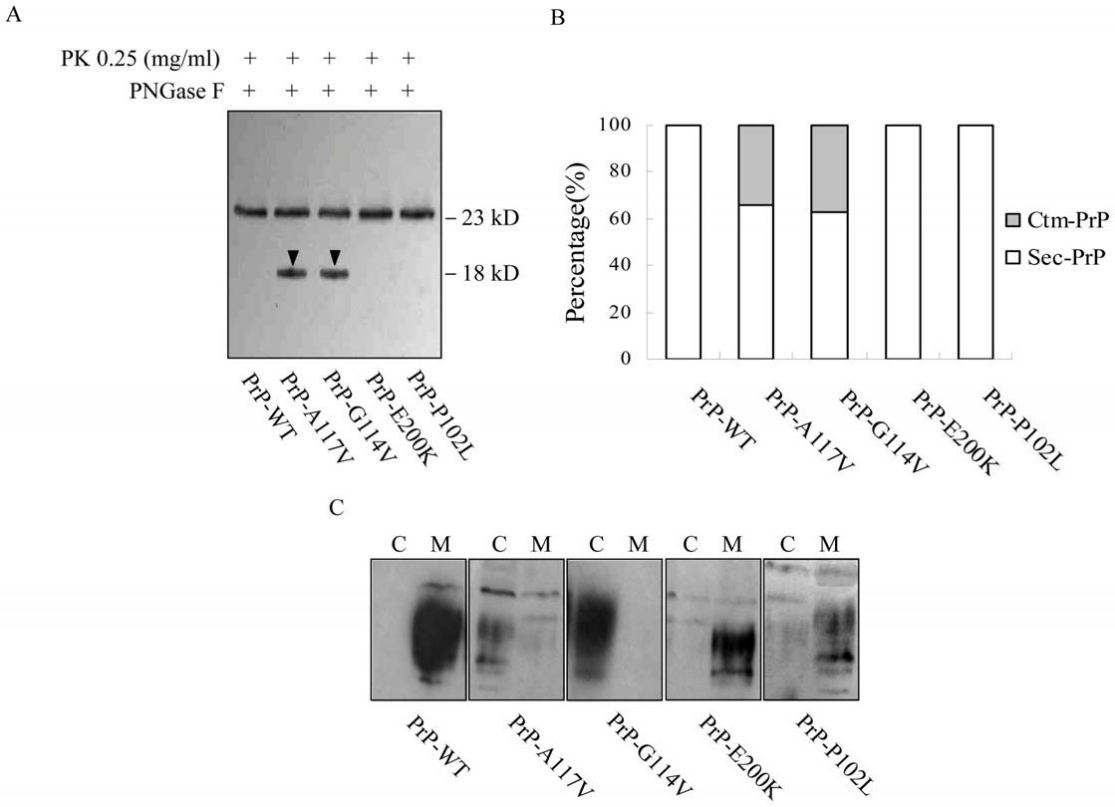
The PrP profiles in the cells expressing various fCJD-associated PrP mutants. A. Formations of Ctm-PrP. The suspended cells were subjected to PK digestion and PNGase F treatment. The arrowheads point to Ctm-PrP. B. Quantitative representation of the relative amounts of Sec-PrP (white bars) and Ctm-PrP (grey bars) for each preparation. C. PI-PLC resistance. Cells expressing WT- and mutated PrP were treated with PI-PLC and PrP signals in the fractions of medium (lanes M) and cellular lysates (lanes C) was detected by Western blots with mAb 3F4.

In order to clarify whether the fCJD-associated PrP mutants in transmembrane region formed the Ctm-PrP, we generated the recombinant plasmids expressing fCJD-related PrP mutants G114V (pcDNA3.1-G114V) and A117V (pcDNA3.1-A117V) within transmembrane region, as well as two other plasmids expressing a GSS-related PrP mutant (P102L, pcDNA3.1-P102L) and an fCJD-related PrP mutant (E200K, pcDNA3.1-E200K) outside the transmembrane (Fig. 1). Western blots of the cell lysates transiently expressing PrP-WT and PrP-mutants, as well as that treated with PNGase F, revealed similar reactive patterns (Fig. S2). Mild proteolysis process followed by PNGase F digestion showed that in addition to the 25 kDa band that presented in all preparation, an 18 kDa PrP-specific band was detected in the preparations of PrP-A117V and PrP-G114V (Fig. 7A), which occupied about 34% and 37% of total PrPs after digitalizing the signal intensities, respectively (Fig. 7B). Expressions of PrP-P102L and PrP-E200K failed to produce such 18 kDa PrP-specific band (Fig. 7A). PI-PLC digestion identified that 24 hr after transfection, most portions of PrP-WT, PrP-P102L and PrP-E200K distributed in the fraction of supernatant, contrarily, large portions of PrP-A117V and PrP-G114V in the pellets (Fig. 7C). These results highlight that under the experimental situation PrP-WT, PrP-P102L and PrP-E200K undergo through the similar process post-translationally and localize on the surface of the cells, whereas PrP-A117V and PrP-G114V locate largely in intra-cytoplasm and formed Ctm-PrP.

### The natural familial CJD associated PrP mutants within transmembrane region led the cells more susceptible to ER stress stimuli

**Figure 8:**
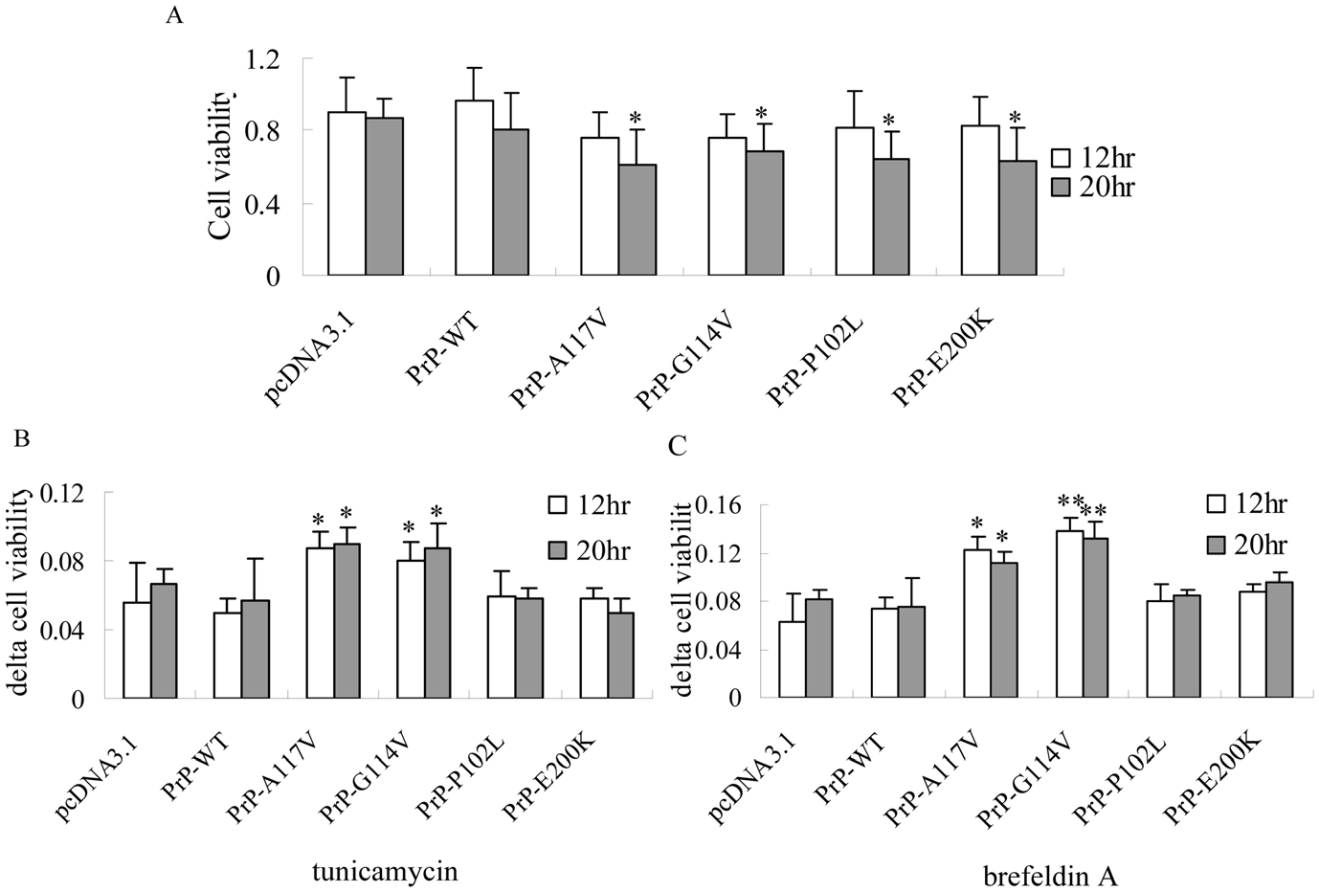
Influences of the ER stress stimuli on the cell viability measured by MTT assays. A. The cell viabilities 12 and 20 hr post-transfection. B and C. The resistant capabilities (delta cell viability) of the cells to tunicamycin (B) and brefeldin A (C). Statistical differences of the data of each preparation compared with that of WT-PrP were illustrated as * *P*<0.05 and ** *P*<0.01, respectively. The average data of each preparation was calculated based on three independent experiments and represented as mean ± SD.

To investigate the influences of various fCJD-associated PrP mutants on the growth abilities of the cultured cells, the cell viabilities were measured with MTT assays 12 and 20 hr after transfection, respectively. No significant difference in the cell viabilities among various groups was addressed 12 hr after transfection (Fig. 8A). However, 20 hr after transfection, all PrP mutants, including PrP-A117V, -G114V, -P102L and -E200K, induced significantly lower cell viabilities compared with PrP-WT and pcDNA3.1 (*P*<0.05, Fig. 8A). It indicates that expressions of all fCJD associated mutants induce cytotoxicity.

To distinguish the possible cytotoxic mechanism of various PrP mutants, cells expressing different PrP constructs were exposed to brefeldin A and tunicamycin and the cell viabilities were evaluated by MTT assays 12 and 20 hr after transfection. It showed that the delta cell viabilities of the cells expressing PrP-A117V and G114V increased obviously in the presences of brefeldin A (Fig. 8B) and tunicamycin (Fig. 8C), revealing significant statistic difference compared with that of pcDNA3.1 (*P<*0.01). Contrarily, expressions of PrP-P102L and PrP-E200K resulted in similar delta cell viabilities as PrP-WT in the presences of brefeldin A and tunicamycin, without statistic difference compared with that of pcDNA3.1 (Fig. 8B and 8C). It suggests that the cytotoxicities due to expression PrP mutants within transmembrane region may largely depend on impairing ER normal function by mislocation.

### The natural familial CJD associated PrP mutants within transmembrane region triggered the ER stress and apoptosis

**Figure 9:**
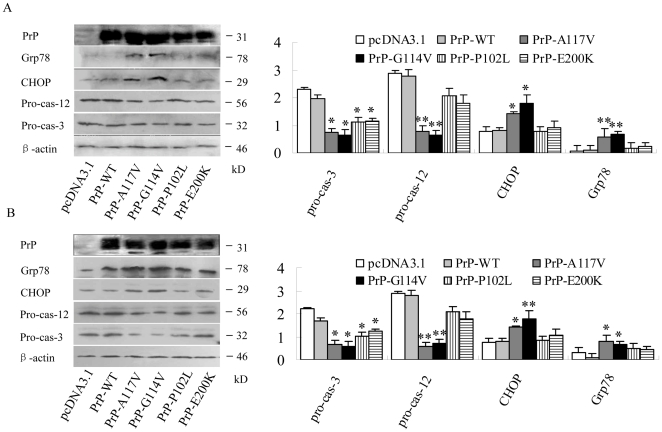
Changes of ER stress- and apoptosis-related events in the cells expressing various fCJD PrP mutants. The levels of Grp78, CHOP, pro-caspase-12 and pro-caspase-3 in SH-SY5Y cells were evaluated by individual Western blots 12 (A) and 20 hr (B) post-transfection. The average gray value of each preparation was calculated by the gray numerical value of each blot vs that of β-actin. The average data of each preparation was evaluated from three independent blots and represented as mean ± SD. Statistical differences of the data of each preparation compared with that of WT-PrP were illustrated as * *P*<0.05 and ** *P*<0.01, respectively.

To detect potential changes of the ER stress related markers after expressions of various PrP mutants, the cellular levels of Grp78, CHOP and pro-caspase-12 protein were evaluated by individual Western blots. It revealed that 12 and 20 hr after transfection, the levels of Grp78 and CHOP in the preparations of PrP-A117V and PrP-G114V were significantly higher than that of either PrP-WT or pcDNA3.1, while that of P102L and E200K increased slightly but without statistic difference (Fig. 9) Meanwhile, the level of pro-caspase-12 in the preparations of PrP-A117V and PrP-G114V decreased remarkably, whereas that of P102L and E200K dropped slightly without statistic difference (Fig. 9). To investigate the change of the down-stream apoptostic protein after expressions of PrP mutants, the cellular level of pro-caspase-3 protein in each preparation was detected by Western blot. and revealed significantly decreased levels in all preparations expressing PrP mutants, compared with that of PrP-WT (Fig. 9). It highlights that although expressions of the tested fCJD-related PrP mutants induce cell apoptosis, they may undergo through different pathways, in which the mutants within transmembrane region are more likely to trigger apoptosis by ER stress process.

## Discussion

In this study, we have provided the data that a novel transmembrane form of PrP tagged with an ER signal peptide (PrP-KDEL) induces the similar biological effectiveness, e.g. formation of Ctm-PrP, ER stress and cell apoptosis, as the previously reported recombinant PrP mutant (PrP-3AV)[9]. We also confirm that retention of full-length presenilin 1 tagged with ER signal peptide (PS1-KDEL) does not induce the ER stress. It supplies the direct evidence that simply retention of PrP in ER will cause subsequent ER stress and cell apoptosis.

Ctm-PrP has the topology of a type II transmembrane protein whose C terminus on the exofacial side of the bilayer. It obeys the “positive inside” rule in which there is a preponderance of positively charged residues on the cytoplasmic side of the membrane-spanning sequence[20]. Most type II proteins contain an internal signal-anchor sequence that serves both to initiate translocation and to anchor the polypeptide chain in the lipid bilayer[21], [22]. A few type II proteins have a uncleaved, N-terminal signal sequence, but unlike the case of Ctm-PrP, this sequence serves as a membrane anchor[23]. The retention of the N-terminal signal peptide on Ctm-PrP can be rationalized by the fact that the N terminus of the polypeptide chain does not enter the ER lumen where signal peptidase is located. In contrast, the signal sequence is cleaved from secretory PrP (Sec-PrP), whose N terminus lie on the lumenal side of the membrane. Ctm-PrP is unusual in the other aspect, which has a C-terminal GPI anchor in addition to the transmembrane anchor. This dual mode of membrane attachment has been described in only a few other proteins[24], [25]. The presence of a GPI anchor on Ctm-PrP is consistent with the fact that anchor addition occurs on the lumenal side of the ER membrane after cleavage of a C-terminal fragment of the polypeptide chain by PI-PLC treatment[26].

Consisting with previous work[27], our study confirms again the hydrophobic domain (residues 112–135) acts as a type II signal-anchor sequence, directing translocation of the C terminus across the membrane to produce Ctm-PrP. Besides the mutations within transmembrane region (3AV and A117V) described before, we prove that another fCJD-related PrP mutant in this region (G114V) forms Ctm-PrP in the transfected cells. Formation of Ctm-PrP seems to be region-specific, since other two fCJD-related PrP mutants (P102L and E200K) outside this region do not produce Ctm-PrP, though these two mutants cause clearly cytotoxicity after expression.

Our results also provide clues of the mechanism by which Ctm-PrP might play a role in the pathogenesis of prion diseases. Hegde et al[9] have hypothesized that Ctm-PrP is a component of a common pathway of neurodegeneration underlying both infectious and genetic forms of prion disease, and that PrP^Sc^ is pathogenic because it enhances the formation of Ctm-PrP[9]. However, the mechanism by which Ctm-PrP may cause neurodegeneration has remained a mystery. Our data emphasize again that Ctm-PrP retained in the ER where it is subjected to proteasomal degradation may damage neurons by activating ER stress-induced signal pathway. Normally, the unfolded protein response (UPR) could results in up-regulation of ER chaperone synthesis, such as Grp78, Grp58, Grp94 and Bip, which are adaptive in nature, but the induction of the transcription factor CHOP/GADD153 and phosphorylation of the translation initiation factor eIF-2 can damage cells by triggering apoptosis[28], [29]. In fact, similar patterns of ER stress, including enhanced level of ER stress up-stream proteins Grp78, Grp58, PERK and Bip, have been specifically observed after formation of Ctm-PrP by expressions of either the genetic engineering recombinant PrPs retained in ER (PrP-KDEL and PrP-3AV) or fCJD PrP mutants within the transmembrane region (PrP-G114V and PrP-A117V), but not by expressions of the fCJD PrP mutants outside the transmembrane region. The presences of the ER stress chaperones trigger the increase of the following ER stress chaperones, CHOP and pro-caspase-12, which may induce apoptosis in a further step.

The exact contribution of Ctm-PrP to the neurodegeneration remains to be determined. However, our study supplies the scientific clues to elicit the different pathways of fCJD PrP mutants for damaging neurons. In line with previous observations, expressions of fCJD PrPs, including point-mutations and inserted mutations in octarepeats, can cause clear cytotoxicity on the cultured cells[30]. Our results indicate that only the mutants within transmembrane region seem to trigger cell apoptosis through ER stress. Interestingly, mutations from wild-type amino acids to valine in this region play essential role in the formation of Ctm-PrP and induction of ER stress. However, the formation of Ctm-PrP seems not directly to associate with the clinical manifestations of fCJD, since CJD-A117V characterizes with ataxia described as a variant GSS and CJD-G114V show clear dementia. Both PrP-A117V mutants containing 129V in previous study[9] and 129 M in this study induce Ctm-PrP. It may imply that, like other CJD subtypes[31], [32], the polymorphism of codon 129 determine the major clinical profiles of the genetic CJD mutated in this region.

We presume that Ctm-PrP may be a component of a common pathway of neurodegeneration underlying both infectious and genetic forms of prion disease, and that PrP^Sc^ pathogenic because it enhances the formation of Ctm-PrP[27]. Both the increase of the levels of Ctm-PrP in mouse brains during the course of scrapie infection and development of a scrapie-like neurological illness without PrP^Sc^ in transgenic mice expressing PrP with Ctm-PrP-favoring mutations[27] emphasize this possibility. ER stress and subsequent apoptosis caused by retention of the abnormal Ctm-PrP in ER may be the normal physiological reaction for cleaning out the misfolding proteins. However, large amounts of accumulation of misfolded proteins in ER will results in irreversible damage on neurons by activating stress-induced signaling pathway.

## Supporting Information

Figure S1 The PrP patterns in the cells transiently expressing PrP-WT, PrP-KDEL or PrP-3AV prior to the treatments of PK and PNGase F (left panel) and digested with PNGase F alone (right panel)in Western blot. Various PrP constructs were indicated at bottom.
(0.93 MB TIF)

Figure S2 The PrP patterns in the cells transiently expressing PrP-WT, PrP-A117V, PrP-G114V, PrP-E200K or PrP-P102L prior to the treatments of PK and PNGase F (left panel) and digested with PNGase F alone (right panel) in Western blot. Various PrP constructs were indicated at bottom.
(1.03 MB TIF)
