# Supplementary figures and images for: Familial CJD Associated PrP Mutants within Transmembrane Region Induced Ctm-PrP Retention in ER and Triggered Apoptosis by ER Stress in SH-SY5Y Cells
Source: PLoS One. 2011 Jan 27;6(1):e14602. doi: 10.1371/journal.pone.0014602 (PMC3029303; doi:10.1371/journal.pone.0014602)

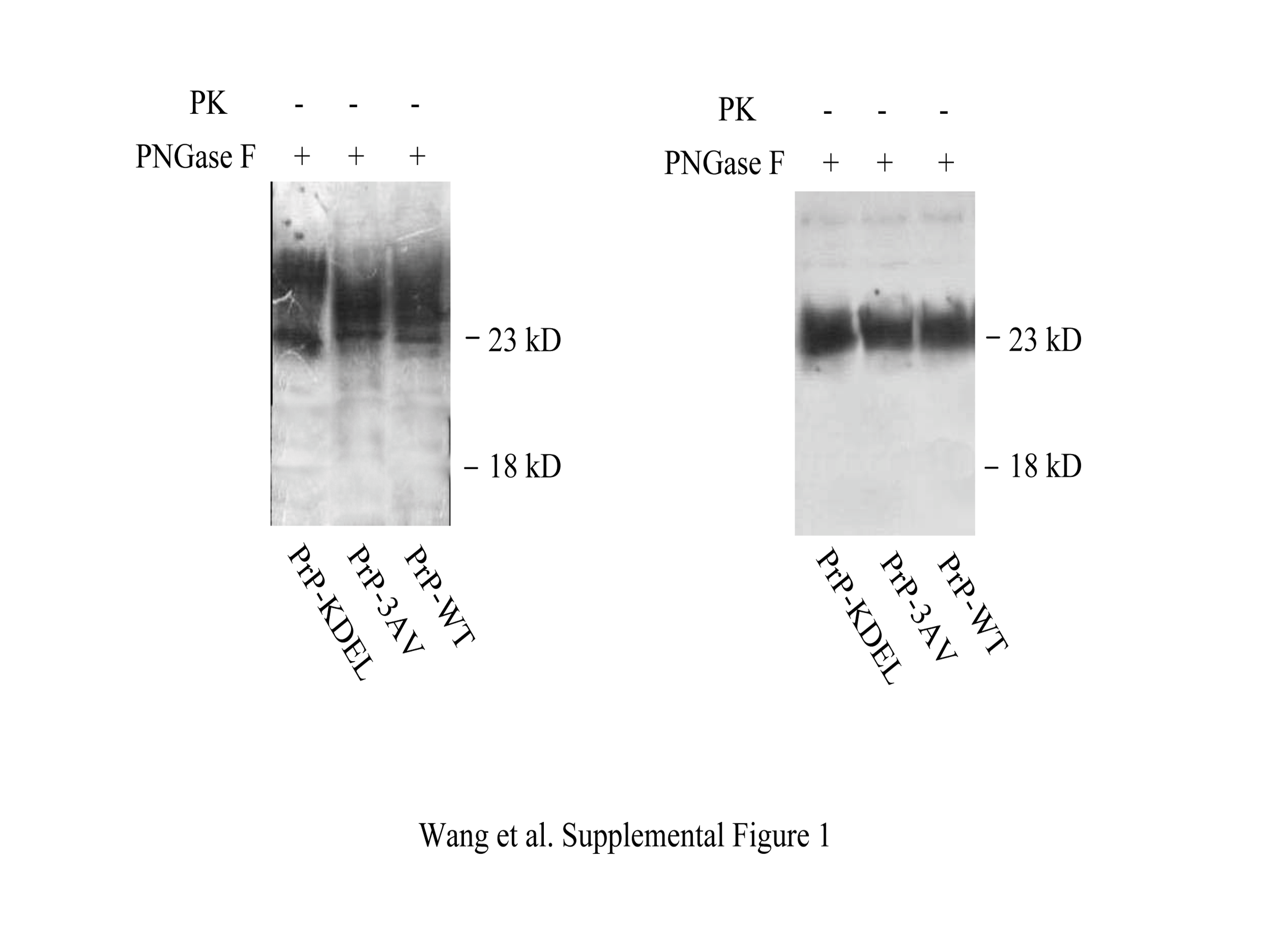

Supplement: Figure S1 — The PrP patterns in the cells transiently expressing PrP-WT, PrP-KDEL or PrP-3AV prior to the treatments of PK and PNGase F (left panel) and digested with PNGase F alone (right panel)in Western blot. Various PrP constructs were indicated at bottom. (0.93 MB TIF) [file pone.0014602.s001.tif]

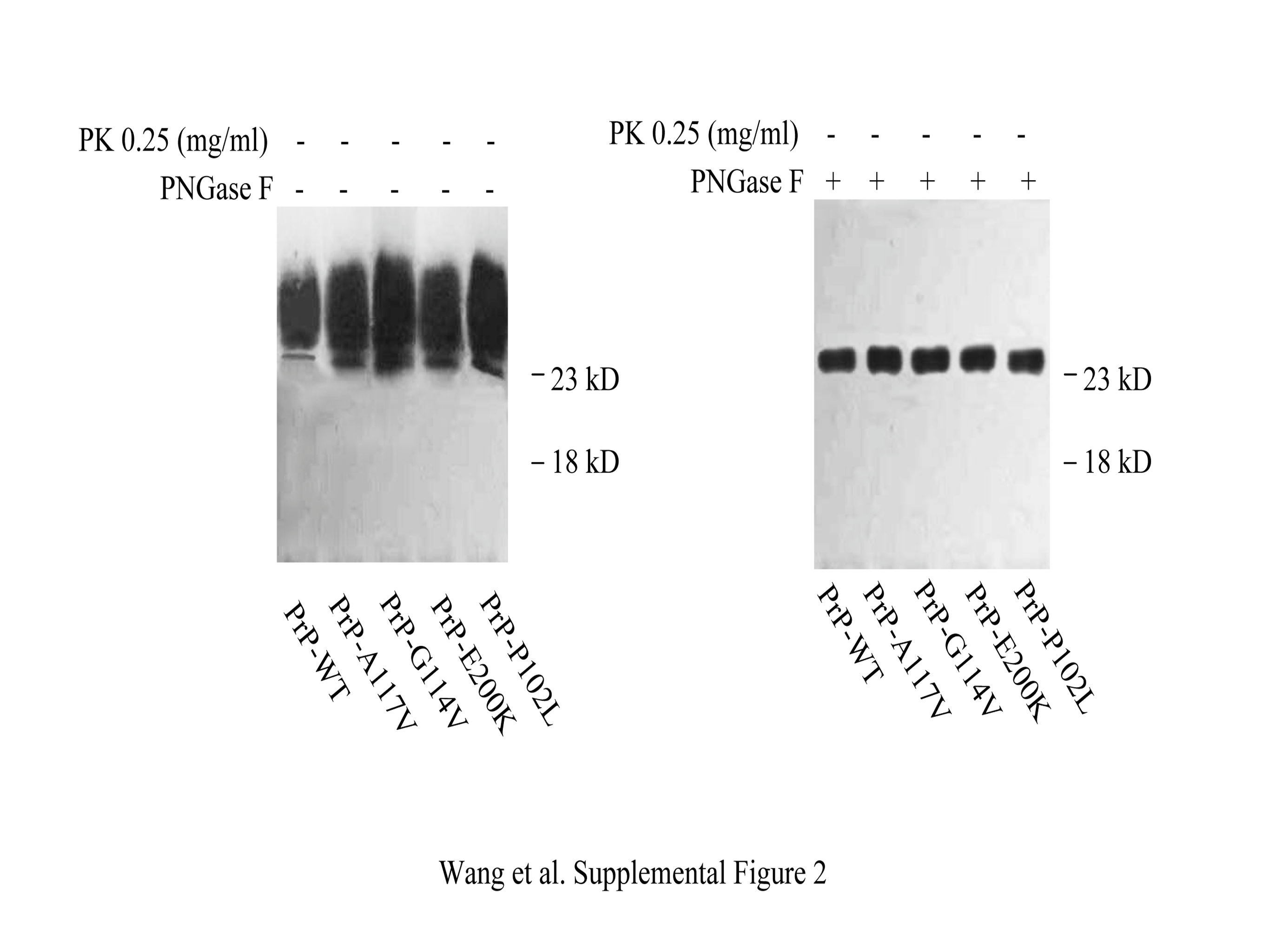

Supplement: Figure S2 — The PrP patterns in the cells transiently expressing PrP-WT, PrP-A117V, PrP-G114V, PrP-E200K or PrP-P102L prior to the treatments of PK and PNGase F (left panel) and digested with PNGase F alone (right panel) in Western blot. Various PrP constructs were indicated at bottom. (1.03 MB TIF) [file pone.0014602.s002.tif]
